# Transdiagnostic efficacy of a group exercise intervention for outpatients with heterogenous psychiatric disorders: a randomized controlled trial

**DOI:** 10.1186/s12888-021-03307-x

**Published:** 2021-06-22

**Authors:** Johanna-Marie Zeibig, Britta Seiffer, Gorden Sudeck, Inka Rösel, Martin Hautzinger, Sebastian Wolf

**Affiliations:** 1 Department of Education & Health Research, Faculty of Economics and Social Sciences, Institute of Sports Science, University of Tuebingen, 72074 Tuebingen, Germany; 2 Faculty of Medicine, Institute for Clinical Epidemiology and Applied Biostatistics, University of Tuebingen, 72074 Tuebingen, Germany; 3 Department of Clinical Psychology and Psychotherapy, Faculty of Science, Psychological Institute, University of Tuebingen, 72074 Tuebingen, Germany

**Keywords:** Exercise, Transdiagnostic efficacy, Depression, Anxiety disorders, Insomnia

## Abstract

**Background:**

Exercise efficaciously reduces disorder-specific symptoms of psychiatric disorders. The current study aimed to examine the efficacy of a group exercise intervention on global symptom severity and disorder-specific symptoms among a mixed outpatient sample.

**Methods:**

Groups of inactive outpatients, waiting for psychotherapy, with depressive disorders, anxiety disorders, insomnia, and attention-deficit/hyperactivity disorders were randomized to a manualized 12-week exercise intervention, combining moderate to vigorous aerobic exercise with techniques for sustainable exercise behaviour change (*n* = 38, female = 71.1% (*n* = 27), *M*_*age*_ = 36.66), or a passive control group (*n* = 36, female = 75.0% (*n* = 27), *M*_*age*_ = 34.33). Primary outcomes were global symptom severity and disorder-specific symptoms, measured with the Symptom Checklist-90-Revised and Pittsburgh Sleep Quality Index pre- and post-treatment. Secondary outcome was the self-reported amount of exercise (Physical Activity, Exercise, and Sport Questionnaire), measured pre-treatment, intermediate-, and post-treatment. Intention-to-treat analyses were conducted using linear mixed models. Linear regressions were conducted to examine the effect of the change of exercise behaviour on the change of symptoms.

**Results:**

The intervention significantly improved global symptom severity (*d* = 0.77, *p* = .007), depression (*d* = 0.68, *p* = .015), anxiety (*d* = 0.87, *p* = .002), sleep quality (*d* = 0.88, *p* = .001), and increased the amount of exercise (*d* = 0.82, *p* < .001), compared to the control group. Post-treatment differences between groups were significant for depression (*d* = 0.63, *p* = .031), sleep quality (*d* = 0.61, *p* = .035) and the amount of exercise (*d* = 1.45, *p* < .001). Across both groups, the reduction of global symptom severity was significantly predicted by an increase of exercise (*b* = .35, *p* = .012).

**Conclusions:**

The exercise intervention showed transdiagnostic efficacy among a heterogeneous clinical sample in a realistic outpatient setting and led to sustained exercise behaviour change. Exercise may serve as an efficacious and feasible transdiagnostic treatment option improving the existing treatment gap within outpatient mental health care settings.

**Trial registration:**

The study was registered on ClinicalTrials.gov (ID: NCT03542396, 25/04/2018).

**Supplementary Information:**

The online version contains supplementary material available at 10.1186/s12888-021-03307-x.

## Background

In 2019, psychiatric disorders affected about 15.6% (point prevalence) of the population in Germany [1]. Psychiatric disorders result in a considerable burden of disease, accounting for 6.4% of disability-adjusted life years (DALYS) [1] and increasing the risk for cardiovascular diseases [2]. The most frequent and burdensome psychiatric disorders are depressive disorders (point prevalence: 4.3%), anxiety disorders (point prevalence: 7.1%), including obsessive compulsive disorders (OCD) and posttraumatic-stress disorder (PTSD), and insomnia (point prevalence: 4%) [1, 3]. Epidemiological studies demonstrated that comorbidity across these disorders is rather the rule than the exception [4]. Additionally, these disorders show a comorbidity with other psychiatric disorders, such as attention deficit hyperactivity disorder (ADHD) [5]. Comorbidity is related to an increased impairment, a worse prognosis and stronger chronicity of symptoms compared to people affected by one single mental disorder [6–8]. The high prevalence and burden of psychiatric disorders as well as their comorbid occurrence, emphasize the substantial need for treating psychiatric disorders. However, in Germany, only 10% of all individuals with psychiatric disorders receive evidence-based treatment and only 2.5% receive psychotherapy [9]. Furthermore, in 2018, the average waiting time for psychotherapeutic treatment was approximately 5 months [10]. The high necessity for treatment, on the one hand, and deficits in mental health care, on the other hand, illustrates the severe gap between people in need for treatment and those actually receiving it [11]. This treatment gap aggravates the burden of psychiatric disorders because the absence or delay in treatment can lead to worsening and chronicity of symptoms and additionally to the development of comorbid diagnoses [10]. Thus, there is a demand for treatment that can improve this treatment gap by being applicable and efficacious to individuals who meet criteria for one or more clinical diagnoses and also for groups of heterogenous disorders. In addition, this treatment would need to be easily and fast accessible (i.e., efficient) to increase the number of people receiving treatment.

The mental health care is dominated by treatments, that are tailored to specific disorders (i.e., disorder-specific treatments) [12] and are usually conducted in individual format rather than in group format [13]. However, considering clinical reality with heterogenous and also comorbid presentations of diagnoses [4], the conduction of those treatments is not efficient because multiple treatment protocols for different specific disorders need to be applied [12]. Furthermore, disorder-specific treatments no longer correspond to recent evidence supposing that underlying shared common etiological and maintenance processes rather than specific diagnoses should be considered when treating psychiatric disorders [12, 14].

To address these underlying mechanisms instead of specific disorders, transdiagnostic psychological treatments, that “[ …] apply the same underlying treatment principles across mental disorders without tailoring the protocol to specific diagnoses” ([26], p.21) have been developed [27, 28, 29, 30, 31]. Recent meta-analyses [27–31], that evaluated the efficacy of transdiagnostic psychological treatments, such as transdiagnostic cognitive behavior therapy (CBT) [27], among patients with depressive disorders and/or anxiety disorders over comparison or control interventions (i.e., diagnosis-specific intervention control, treatment-as-usual, or a waitlist control), demonstrated moderate to large effect sizes. This suggests that transdiagnostic treatments can efficaciously augment dominating disorder-specific treatments [12].

There is a body of evidence assuming that exercise, defined as physical activity that is planned, structured, and repetitive, with the primary aim to improve or maintain physical fitness [32] might represent a potential transdiagnostic treatment for depressive disorders, anxiety disorders, insomnia and ADHD. Findings of a recent meta-analysis [33] investigating the effects of exercise on underlying processes of these psychiatric disorders (i.e., anxiety sensitivity, distress tolerance, stress reactivity, and general self-efficacy) revealed a large effect of exercise on reducing anxiety sensitivity, a moderate effect on increasing general self-efficacy and a small effect on reducing stress reactivity. Similarly, a recent meta-analysis of randomized controlled trials (RCTs) [34], which examined the impact of exercise on sleep quality among people with psychiatric disorders, revealed a large beneficial effect on the improvement of sleep quality. Supporting the assumption of the transdiagnostic efficacy of exercise, results of numerous systematic reviews and meta-analyses have suggested moderate to large disorder-specific effects of exercise on depressive disorders [35, 36], anxiety disorders [37, 38], insomnia [39, 40] and ADHD [41, 42] over non-active [37, 39], or active and non-active controls [35, 36, 38, 40, 41].

Exercise conducted two to three times per week, for approximately 10 weeks, at a minimum of moderate intensity and a duration of 30 min, partially supervised or non-supervised, solely aerobic or aerobic combined with resistance training, are key components of previous disorder-specific exercise interventions and seem to be associated with optimal therapeutic efficacy among patients with specific disorders [33, 35,37, 43]. These beneficial effects, such as the reduction of depressive symptoms, seem to be only sustainable when exercise is conduced and maintained on a regular base. In an exercise trial [44], the antidepressant effect of exercise was compared to those of psychopharmacotherapy in patients with depressive disorders. Both conditions showed similar improvements in depressive symptoms. In the one-year follow up study, however, this effect was only maintained for those participants who engaged in exercise on a regular base [45]. Exercise interventions combined with behavior change techniques (BCTs), that increase motivation and volition for exercise seem to increase the initiation and maintenance of exercise among healthy and psychiatric samples [46, 47, 48, 49].

Despite of the promising evidence of the efficacy of exercise among specific disorders and underlying processes across disorders, to the best of our knowledge, the effects of exercise have never been investigated across a broad range of diagnostically heterogeneous and a highly comorbid clinical sample in a realistic outpatient setting. Furthermore, as far as we know, there is no exercise trial that includes heterogenous disorders and assessed global symptom severity with a reliable and clinical measure.

Therefore, the manualized group exercise intervention, named “ImPuls” [50], was developed. The intervention was developed for inactive outpatients, waiting for psychotherapeutic treatment in German health care settings in outpatient units and practices, suffering from one or more diagnoses of depressive disorders, anxiety disorders, insomnia, and ADHD. The exercise intervention integrates the most recent findings about optimal modalities of exercise for therapeutic efficacy for the targeted disorders and sustainable behavior change by integrating behavior change techniques (BCTs). All components of this intervention are tailored to the care reality of outpatients with psychiatric disorders to offer an efficient treatment option in the outpatient mental health care setting: 1) including a broad range of heterogenous diagnoses for which prior research has demonstrated therapeutic efficacy, 2) conducting the intervention in group format, 3) short duration (i.e., 4 weeks of supervised sessions, 8 weeks non-supervised exercise) to be feasible during waiting times for psychotherapy.

The primary hypothesis was that participants of the intervention group would demonstrate lower global symptom severity and disorder-specific symptoms (depression, anxiety, sleep quality) at post-treatment assessment compared to a passive control group. The secondary hypothesis was that participants of the intervention group would demonstrate a significant larger amount of exercise at intermediate- and post-treatment assessment compared to the control group. It was further hypothesized that the increase of the amount of exercise from pre- to post-treatment assessment would predict the reduction of global symptom severity and disorder-specific symptoms from pre- to post-treatment assessment across both treatment arms.

## Methods

### Study design

The study was conducted at the University of Tuebingen. Active enrollment lasted from April 2018 to October 2019. The study was registered on ClinicalTrials.gov (ID: NCT03542396, 25/04/2018) and approved by the local ethics committee for psychological research (Az_Wolf_2018_0108_99). A block-randomized (allocation ratio 1:1) parallel trial with two treatment arms (intervention group, control group) and three measurement points (pre-, intermediate-, post-treatment assessment) was conducted. Primary outcomes were assessed at pre- and post-treatment assessment; secondary outcome at pre-, intermediate- and post-treatment assessment. The study was reported according to the Consolidated Standards of Reporting Trials (CONSORT) statement [51].

### Recruitment and participants

Recruiting was performed at the outpatient clinic of the University of Tuebingen, medical and psychotherapist’s offices, and by media advertising. Inclusion criteria were age between 18 and 65 years, fluent in German, no medical contraindications for exercise (participants needed to receive a medical consultation prior to the intervention that confirmed participant’s ability to exercise), on a waiting-list for outpatient psychotherapy and diagnosed according to DSM-IV-TR with at least one of the following disorders: depressive disorders (F32, F33, F34.1), anxiety disorders (F40.0, F40.1, F40.2, F41.0, F41.1, F41.2, F42, F43.1, insomnia (F51.0), and ADHD (F90.0, F90.1, F98.8). Exclusion criteria included: acute substance use disorders (F10.2, F11.2, F12.2, F13.2, F14.2, F15.2, F16.2, F18.2, F19.2), chronic pain disorder (F45.1), eating disorders (lifetime; F50), bipolar disorder (lifetime; F31), antisocial personality disorder (F60.2), borderline personality disorder (F60.3), acute suicidal tendencies, regular exercise (≥ 30 min/week), change (i.e., reduction and increase) of psychopharmaceuticals (≤ 2 months). Any change in treatment led to a withdrawal from the study.

The sample size was determined a-priori through power analysis, using G*Power ([52], v. 3.1.9.7.). A moderate to large effect (Cohen’s *d* = 0.74) of the mean post-difference between the two groups for the primary outcome was expected. Effect estimation was the median of the effect of exercise on disorder-specific symptoms of depressive disorders, anxiety disorders and insomnia. Since a small number of eligible participants with ADHD was expected, the effect of exercise on symptoms of ADHD was not included in the effect estimation. Effect sizes were derived from the most recent meta-analyses and RCTs available at the time of trial design [36, 40, 53]. With significance level: *α* = .05, Power: *1-β* = .80, allocation ratio N2/N1: *ε* = 1, the required sample size for a two-tailed t-test for independent samples was *N* = 60. With an expected dropout rate of 18% for a clinical sample in an RCT [38], the total sample resulted in *N* = 71. This resulted in a group size of approximately *n* = 36 for each group.

### Randomization

Randomization was performed when the maximum of participants for one treatment group (*n* = 5–10) was eligible and/or when remaining waiting time for psychotherapy was shorter than intervention period (≤3 months). Participants were assigned a code, which was sent to a research assistant. They were randomly assigned to a group, based on a randomization table, stratified by age and symptom severity (Global Assessment of Functioning Scale, GAF) for each group, using MATLAB (9.6.0, R2019a). The study therapist received the allocation information, matched the codes to participants and informed them of their group allocation.

### Procedure

Psychologists performed a preliminary telephone screening of eligibility criteria. Eligible participants were invited to the University of Tuebingen where they first completed a demographic questionnaire. Research assistants, who were trained in the conduction of structured clinical interviews, conducted the structured clinical interview for DSM-IV (SCID) [54] for eligibility criteria. ADHD was diagnosed by DSM-IV criteria through the Homburg ADHD Scales for Adults (HASE) [55]. Primary insomnia was diagnosed by DSM-IV criteria combined with the Pittsburgh Sleep Quality Index (PSQI) [56]. Participants signed an informed consent form and completed online questionnaires via a secure online survey software Sosci Survey [57] prior to randomization. This *pre-treatment assessment* was conducted on an average of 23.15 days (*SD* = 22.19) prior to the start of the intervention.

Ten groups of five-to-ten participants were sequentially allocated to the intervention group or control group. The intervention group completed the exercise intervention, while the control group did not receive any treatment. At week 9 of the intervention (*intermediate-treatment assessment*), participants’ amount of exercise was assessed. No other assessments were conducted at this assessment point. Intermediate-treatment assessment was scheduled at week 9 of the intervention because it was middle of the non-supervised time. The non-supervised intervention period started at week 5 of the intervention and lasted 8 weeks in total.

One-to-two weeks after intervention (*post-treatment assessment*), the same procedure of the pre-treatment assessment was performed. The SCID was also conducted again at post-treatment assessment by an outcome assessor (research assistants, who were trained in the conduction of structured clinical interviews) that was blind to group assignment. Afterwards, the control group received 50€ as a compensation for their time. All participants were offered preferential psychotherapy at the outpatient clinic in Tuebingen after post-treatment assessment. The final post-treatment assessment was conducted in October 2019.

### Assessments

#### Primary outcomes

##### Global symptom severity

Global symptom severity was measured by the Global Severity Index (GSI) of the German version of the self-reported questionnaire Symptom Checklist-90-Revised (SCL-90-R [58];). The GSI comprises the average distress rating on all symptom scales and ranges from 0 to 4. Higher scores indicate higher distress. Clinically relevant changes are defined as a change on the GSI of at least 0.26 [58]. Among patients with affective disorders, the GSI has demonstrated good internal consistency (*α* = .97) and construct validity (*r* = .77) [59]. The internal consistency in the current study was *α* = .97 for the GSI.

##### Depression and anxiety

Depression and Anxiety were measured by the sub scores Depression and Anxiety of the self-reported questionnaire SCL-90-R [58]. Items of the depression sub score ask participants for symptoms of depression (e.g., “How much did you suffer from a decrease in your interest or pleasure in sexuality in the past few days?”) and items of the anxiety sub score for symptoms of anxiety (e.g., “How much did you suffer from nervousness or inner trembling in the past days?”). Scales ranges from 0 to 4 with higher scores indicating higher distress. Symptoms are clinically raised when T-values are ≥60. Among patients with affective disorders, the depression scale (*α* = .89) and anxiety scale (α = .87) have demonstrated internal consistency and construct validity (*r* = .80 for depression and *r* = .61 for anxiety) [59]. The internal consistency in the current study was *α* = .91 for depression and *α* = .90 for anxiety.

##### Sleep quality

Sleep Quality was measured by the global sleep quality score of the German version of the self-reported questionnaire Pittburgh Sleep Quality Index (PSQI [56];). The global sleep quality score is the sum of seven sleep component scores (range of component scores: 0–3): subjective sleep quality (“Over the last four weeks, how would you rate your overall sleep quality?”), sleep latency (e.g., “Over the last four weeks, how long have had it usually take you to fall asleep at night?), sleep duration (e.g., “Over the last four weeks, how many hours have you actually slet per night?”), habitual sleep efficiency (e.g., “Over the last four weeks, what time have you usually gotten up in the morning?”), sleep disturbances (e.g., “Over the last four weeks, how often have you had a bad night’s sleep because you couldn’t fall asleep within 30 minutes?”), use of sleeping medications (e.g., “Over the last four weeks, how often have you taken sleeping pills (prescribed by a doctor or over-the-counter)?”), and daytime dysfunction (e.g., “Over the last four weeks, how often have you had difficulty staying awake, such as while driving, eating, or attending social events?”). The global sleep quality score can vary from 0 to 21 with a cut-off score of 5, identifying clinically raised sleep impairment [56]. It has shown a high sensitivity (98.7%) and specificity (84.4%) in identifying insomnia [60]. In the literature, the internal consistency for the global sleep quality score was *α* = .77 [61]. In the current study, it was *α* = .67.

#### Secondary outcome: exercise

The amount of exercise was measured using the self-reported exercise activity index of the physical activity, exercise, and sport questionnaire (BSA questionnaire [62];). Participants specify whether they have engaged in regular exercise in the past 4 weeks (“Have you engaged in regular exercise in the past 4 weeks?”). If they have engaged in regular exercise, participants were asked to specify the type (“What kind of exercise activity (ies) have you engaged in?”), frequency and duration (“I have engaged in activity x approximately … times in the past four weeks, every time for approximately … minutes”) of it. The exercise activity index indicates the average minutes of exercise per week. The BSA questionnaire can validly assess changes in exercise behavior. Data of reliability is not available [62] and could not be analyzed for the current study it because this scale consists of one item only.

#### Further assessments

Further measures (self-reported questionnaires), described in the trial registration, but not included in the current evaluation, were the Sleep Questionnaire B-Revised version (SF-B [63];), Perceived Stress Scale (PSS [64];), 36-Item Short Form Health Survey (SF-36) (SF-36 [65];), Self Concordance of Sport- and Exercise-related Goals Scale (SKK-Scale [66];), Scales that assess motivation and volition for exercise [67], and Physical Activity-related Health Competence Questionnaire [68]. Accelerometer derived exercise at seven consecutive days was assessed. 5-min resting heart rate variability was assessed by a trained research assistant.

### Exercise intervention

**Fig. 1:**
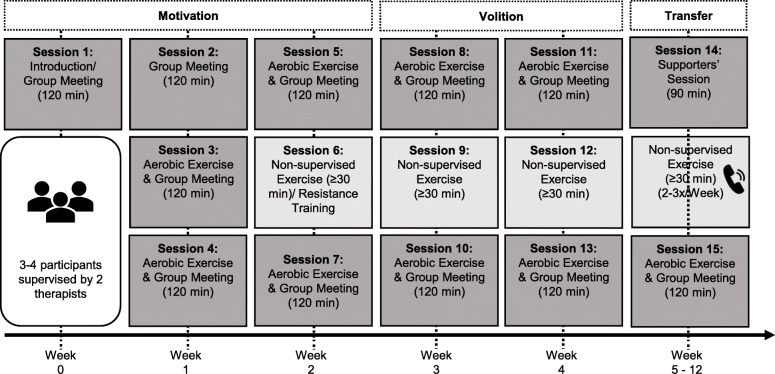
Intervention procedure of the group exercise intervention. Dark grey boxes represent group meetings combined with supervised aerobic exercise (starting session 3) and the supporters’ session (session 14 in week 5). Session 1 contains an introduction into the intervention. Session 15 is conducted in week 12. Light grey boxes represent non-supervised exercises or resistance training, which can be conducted as participants’ first non-supervised exercise (session 6). The phone represents the weekly phone contact from week 5–12. Week 1–2 mainly aimed to increase motivation, week 2–4 mainly increase volition. Week 5–12 aimed to transfer motivation and volition for exercise into participants’ daily life routines

The 12-week exercise intervention “ImPuls” [69] was conducted in a group format of three to four participants and divided into a supervised and non-supervised period. Behaviour change techniques (BCTs), such as self-efficacy, goal setting, self-monitoring, formation of concrete exercise plans and coping planning, were integrated to promote sustained exercise behaviour change [49, 70]. Intervention contents are displayed in Fig. 1 and Table 1.

**Table 1:** Overview of behaviour change techniques of the group exercise intervention

Focus	Technique
Motivational (mainly week 1–2)	Education about positive and negative effects of exercise
	Education about optimal modalities of exercise to experience positive psychological effects
	Selection of a preferred activity
	Goal setting
	Self-monitoring of goal achievement
	Reflection about positive experiences/effects with/of exercise
Volitional (mainly week 3–4)	Identification of barriers to exercise
	Techniques to overcome barriers
	Social support (intervention group and trainers)
	Exercise self-monitoring (optimal modality)
Motivational and volitional (week 5–12)	Social support (family, friends, trainer)
	Self-monitoring of goal achievement
	Exercise self-monitoring (optimal modality)

#### Supervised period (week 0–4)

Participants took part in a combination of supervised running session and group meetings with a total duration of 120 min. One supervised session is conducted during week 0 (introduction to the intervention), three supervised sessions during the second week, two supervised sessions plus one non-supervised exercise during week 2–4, respectively. Running lasted 30 min and participants could choose a standardized interval-based training or endurance training. Both training methods were conducted with moderate intensity, which was tracked by a heart rate monitor (Polar Electro GmbH; A 300) combined with a chest strap (Polar Electro GmbH; H7) and the Borg Rating of Perceived Exertion (RPE) Scale [71]. Moderate intensity was defined as 60 to 80% of maximum heart rate, subtracting age from 220 [72] and from nine to 14 on the RPE Scale [71]. Participants engaged in additional 30-min, moderate-intensity, non-supervised exercise.

#### Non-supervised period (week 5–12)

During the non-supervised period, participants engaged in 30-min, moderate-intensity, non-supervised exercise two to three times a week, which was accompanied by an activity diary and weekly phone calls with one study therapist, intending to maintain motivation, volition, and adherence to exercise. A session for participant’s supporters (e.g., friends, partner) was scheduled in week 5 to inform them about the possibilities to aid the participants in transforming their intentions into action. A final group meeting with a running session took place in week 12 in order to exchange experiences and to encourage participants to maintain regular exercising.

#### Attendance and adherence

Participants performed 30 min non-supervised exercise if they missed a supervised running session. Contents of group meetings were repeated in one-to-one sessions (by phone or face-to-face). The assessment of the amount of exercise (see Procedure) served as a measure of adherence during the non-supervised period (see Table 3).

#### Therapists

All main therapists were master’s degree psychologists with either fully approved in psychological psychotherapy or in advanced postgraduate training for CBT. Assistant therapists were graduate students of psychology. All therapists were trained in the background and contents of ImPuls und supervised by an exercise therapist and a psychotherapist.

#### Potential risks and benefits of the intervention

Most common risks associated with exercise are cardiovascular events and musculoskeletal injuries [73]. Intermediate and long-term potential benefits of exercise are improved physical health, such as prevention of diabetes (e.g., [74]) and improved mental health, such as the reduction of depressive symptoms (e.g., [75]). Furthermore, majority of individuals are exposed to greater risk by not exercising. Indeed, sedentary behavior seem to be associated with a higher risk to develop mental disorders [76, 77] and reduced physical health [78]. Since all participants of the current intervention needed to receive a medical consultation prior to the intervention, the potential risk of exercise on physical health was minimized. Therefore, potential benefits seemed to outweighs the risks of participation in the study.

### Data diagnostics

Individuals from both study arms, who fulfilled any exclusion criteria (i.e., change of treatment, contraindications for exercise) during the intervention, were excluded from post-data collection, 18.1% (*n* = 13). Since the current study contained exclusion criteria that could change during study participation (e.g., change of treatment), data from participants, who did no longer meet eligibility criteria needed to be excluded from post-data collection in order to reduce the risk of bias in the intervention effect estimate [79]. Missing data was assessed at scale level. Linear mixed models, using maximum likelihood estimations, were conducted to handle missing data (also participants’ data of those who were excluded from post-data-collection). Linear mixed models, based on all observed data, can be a valid and unbiased method to handle data that is missing at random [80].

Potential outliers were identified through three measures: Leverage, Cook’s Distance, and Studentized Residuals. Cases with greater than three time the average leverage, cook’s distance greater than 1, and studentized residuals greater than 3 [81, 82], were considered as potential outliers or influential data points. To evaluate the influence of potential outliers on results of linear mixed models, they were calculated again without cases that either exceeded the cut-off value of all three measures, or exceeded the cut-off values of cook’s distance and leverage, or exceeded the cut-off value of cook’s distance. Post-difference and interaction-effect sizes were then compared to those of linear mixed models including the complete data set.

Assumptions of linear mixed models and linear regressions (i.e., linearity, normality of the residuals, homoscedasticity, and multicollinearity) were visually inspected. If residuals of linear mixed models were not normally distributed, data was log-transformed.

### Analytic strategy

For data preparation the Statistical Package for Social Science (IBM SPSS, Inc., Chicago, IL, USA, version 26) was used. Statistical analyses were carried out using R (version 4.0.3) and RStudio (version 1.1.453). Descriptive statistics were used to analyze sample characteristics and dropout rates. Baseline differences between groups and between those that completed the study versus dropped out were analyzed using two-tailed t-tests for independent samples (continuous variables), Chi-scared tests or fisher’s exact tests (categorical variables).

Differences between groups on primary and secondary outcomes changes were analyzed as intention-to-treat (ITT) analysis with linear mixed models using the lme4 package [83] in R. Models used maximum likelihood as the estimation technique. Models included *treatment group*, *time point* and *time point-by-treatment group* interaction as fixed effects. Intercepts were included as random effects. An unstructured covariance matrix was assumed. Post-hoc tests for post-treatment-differences between groups were carried out using two-tailed t-test for independent samples. For post-hoc tests of the secondary outcome, a Bonferroni correction was used. Effect sizes for differences between groups (intermediate-, post-treatment-difference effects and interaction effects) were calculated according to Cohen’s d [84].

An additional analysis inspected clinically relevant changes of participants’ symptomatology. Clinically relevant changes were defined as a change on the GSI from pre-treatment to post-treatment assessment of at least 0.26 [58].

Sufficient statistical power enabled exploratory analyses that examined the efficacy of exercise on depression for subgroups of participants diagnosed with depressive disorders. Additional exploratory analysis (per-protocol analysis) tested the predictive value of the change of the amount of exercise on primary outcomes changes. Linear regressions were calculated with change of exercise (post-treatment assessment – pre-treatment assessment) as a predictor variable and primary outcomes changes (pre-treatment assessment – post-treatment assessment) as criterion variables.

## Results

### Participant flow during the study

**Fig. 2:**
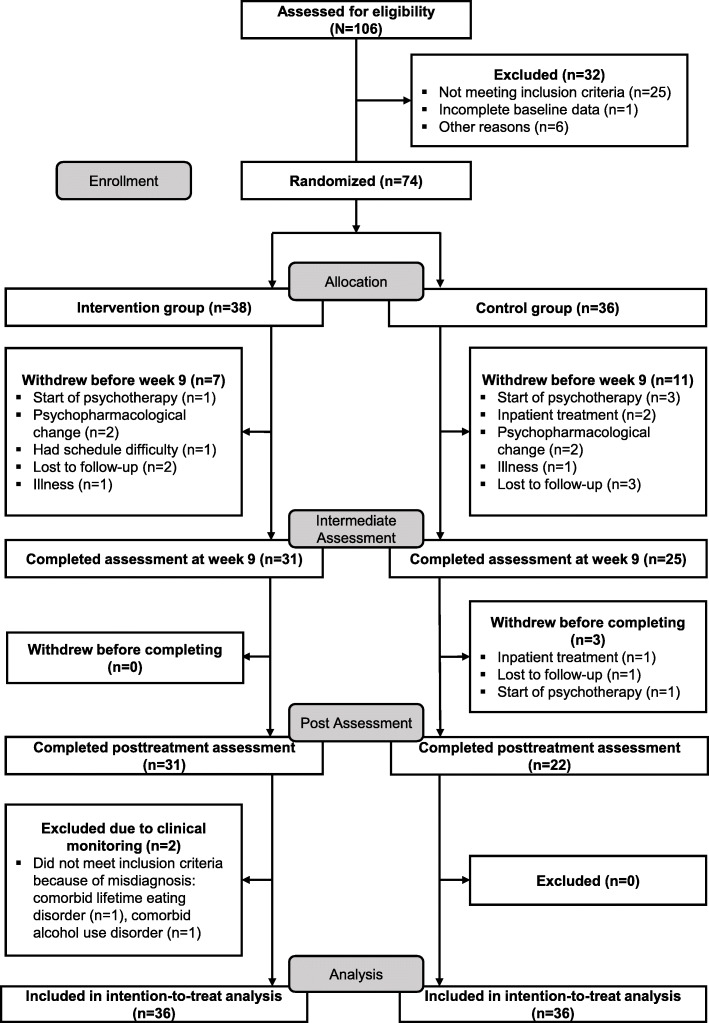
CONSORT flow diagram: profile and enrollment and flow through the randomized controlled trial of group exercise intervention versus a passive control group for individuals with mental disorders

In total, 106 individuals were assessed for eligibility. Of those assessed, 30.2% (*n* = 32) were excluded and 69.8% (*n* = 74) were eligible for participation. Those eligible were randomly assigned to the intervention group, 51.3% (*n* = 38), or control group, 48.7% (*n* = 36). 5.3% (*n* = 2) of participants of the intervention group were removed from the analysis due to a wrong diagnosis at pre-treatment assessment (comorbid lifetime eating disorder, 2.6% (*n* = 1), and a comorbid alcohol use disorder, 2.6% (*n* = 1) [79]. This led to an ITT efficacy group of *N* = 72 (50.0% (*n* = 36) of patients in the intervention group vs. 50.0% (*n* = 36) in the control group) (see Fig. 2).

### Participant characteristics at baseline

Baseline characteristics of the studied sample are reported in Table 2. There were no significant baseline differences between intervention group and control group. Dropout rates of the control group were higher compared to the intervention group, but not statistically different. Compared to the intervention group, participants of the control group dropped out more frequently due to treatment change without reaching statistical significance (see Table 2 and Fig. 2). There were no significant baseline differences between study completers and dropouts (Additional file 1). There were no serious adverse events.

**Table 2:** Baseline demographic and clinical characteristics of participants

	Intervention Group		Control Group		Intervention Group Compared With Control Group (***N*** = 72)		
	(***N*** = 36)		(***N*** = 36)				
Measure	N	%	N	%	χ^2^	df	*p*
Female	25	69.4	27	75.0	0.28	1	.599
Married or partnered	25	69.4	24	66.7	0.06	1	.801
High school	31	86.1	25	69.4	2.89	1	.089
Employment^a^	33	94.3	31	88.6			.673^h^
Diagnosis							.866^h^
Depressive disorders (single)^b^	13	36.1	11	30.6			
Depressive disorders (comorbid with anxiety disorders)^c^	10	27.8	13	36.1			
Panic disorder	2	5.6	2	5.6			
Social anxiety disorder	2	5.6	2	5.6			
Specific anxiety disorder	2	5.6	0	0.0			
Generalized anxiety disorder	1	2.8	0	0.0			
Agoraphobia	1	2.8	1	2.8			
Obsessive-compulsive disorder	0	0.0	0	0.0			
Obsessive-compulsive disorder and social anxiety disorder	0	0.0	2	5.6			
Post-traumatic stress disorder	1	2.8	2	5.6			
Primary insomnia	3	8.3	3	8.3			
Attention deficit hyperactivity disorder	1	2.8	0	0.0			
Clinically raised symptoms (SCL-90-R, PSQI)	36	100.0	34	94.1			.493^h^
Depression^d^, anxiety^d^, insomnia^e^	21	58.3	17	47.2	0.89	1	.345
Receiving psychiatric drugs^f^	18	50.0	20	57.1	0.36	1	.546
Dropout	7	19.4	14	38.9	3.29	1	.070
Treatment change^g^	3	8.3	9	25.0			.111^h^
	Mean	SD	Mean	SD	t	df	p
Age (years)	37.33	14.23	34.33	12.39	0.95	68.69	.343
Global Severity Index (SCL-90-R)	1.06	0.63	1.02	0.61	0.27	69.91	.790
Sleep Quality (PSQI)^e^	9.67	3.66	8.62	3.15	1.28	68.04	.204
Latency pre-treatment – initiation (days)	21.94	17.21	24.36	26.44	−0.46	60.15	.648

*Note*. *SCL-90-R* Symptom Checklist-90-Revised, *PSQI* Pittsburgh Sleep Quality Index

^a^ For employment, the number of participants was *n* = 35 for the intervention group and *n* = 35 for the control group because of incomplete data at pre-treatment assessment.

^b^ Depressive disorders (single) includes all participants diagnosed with a single depressive disorder.

^c^ Depressive disorders (comorbid with anxiety disorders) includes all participants diagnosed with depressive disorder and comorbid anxiety disorders.

^d^ Depression and anxiety levels were classified as clinically raised when t-values of the sub-scales depression and anxiety of the SCL-90-R were greater or equal to 60 (reference group: healthy men, aged between 35 and 44).

^e^ Symptoms of insomnia were classified as clinically raised when the cut-off value of five of the total sum score of the PSQI were reached. For clinically raised symptoms and sleep quality, the number of participants was 36 for the intervention group and 35 for the control group because of incomplete data at pre-treatment assessment on the PSQI.

^f^ For receiving psychiatric drugs, the number of participants was *n* = 36 for the intervention group and *n* = 35 for the control group because of incomplete data at pre-treatment assessment.

^g^ Treatment change is defined as the start of psychotherapy, inpatient treatment or psychopharmacological change.

^h^ Fisher’s exact test was used as a replacement for the chi-square test because the frequency of one or more cells was less than 5

### Statistics and data analysis

Missing data was missing completely at random (MCAR) (*χ*^*2 *^(14) = 12.33, *p* = .580). 106 (14.7%) values of primary and secondary outcomes were incomplete. Per-protocol analyses for each primary and secondary outcome were conducted as sensitivity analyses, using linear mixed models (Additional file 2), to examine the robustness of the results.

Ten cases were detected as potential outliers in the linear mixed model with the GSI (SCL-90-R) as criterion variable and four cases in the linear mixed model with the global sleep quality score (PSQI) as criterion variable. No cases were detected as potential outliers in the mixed models with exercise (BSA questionnaire) as criterion variable. Excluding potential outliers did not change direction, nor significance of intervention effects (Additional file 4). Intervention effects on the global sleep quality score increased from moderate to large effect size (cohen’s d for post-treatment-difference between groups) when potential outliers were excluded. Therefore, potential outliers were not excluded from analyses.

### Efficacy of the intervention

Results of all primary and secondary outcomes and subgroup analysis, including effect sizes, are presented in Table 3. The interaction effect of group by time was significant for global symptom severity, *F* (1, 53) = 7.93, *p* = .007, 95%CI[− 0.50,- 0.08], depression, *F* (1, 55) = 6.30, *p* = .015, 95%CI[− 0.82, − 0.09], anxiety, *F* (1, 54) = 10.12, *p* = .002, 95%CI[− 0.36, − 0.08], and sleep quality, *F* (1, 58) = 11.24, *p* = .001, 95%CI[− 4.68, − 1.19], with larger decreases in the intervention group. Post-treatment difference effects were significant for depression, *t* (114) = 2.18, *p* = .031, and sleep quality, *t* (121) = 2.13, *p* = .035, with lower scores in the intervention group. Post-treatment difference effects for global symptom severity, *t* (105) = 1.68, *p* = .096, and anxiety, *t* (110) = 1.88, *p* = .063, were moderate, with lower scores in the intervention group, without reaching significance.

**Table 3:** Marginal means, confidence intervals, effect sizes, and results of linear mixed models analyses

	**Intervention Group** **(*N*** **= 36)**			**Control Group** **(*N*** **= 36)**				**Change from Baseline in Intervention Group Compared With Control Group (*N*** **= 72)**		
**Measure and Assessment Point**	**Mean**	**SD**	**95% CI**	**Mean**	**SD**	**95% CI**	**d**^**a**^	**B**	**95% CI**	**d**^**b**^
Global Severity Index (SCL-90-R)								−0.30	−0.50,-0.08	0.77**
Pre-treatment	1.06	0.59	0.87,1.26	1.02	0.59	0.83,1.22				
Post-treatment	0.67	0.55	0.47,0.87	0.93	0.51	0.71,1.14	0.48			
Depression (SCL-90-R)								−0.46	−0.82,-0.09	0.68*
Pre-treatment	1.57	0.85	1.29,1.85	1.60	0.85	1.32,1.88				
Post-treatment	0.94	0.80	0.65,1.24	1.43	0.76	1.11,1.76	0.63*			
Anxiety (SCL-90-R)^c^								−0.22	−0.36,-0.08	0.87**
Pre-treatment	0.60	0.35	0.49,0.72	0.56	0.35	0.44,0.67				
Post-treatment	0.36	0.33	0.24,0.48	0.53	0.31	0.40,0.67	0.53			
Sleep Quality (PSQI)								−2.94	−4.68,-1.19	0.88**
Pre-treatment	9.67	3.31	8.57,10.76	8.67	3.29	7.57,9.77				
Post-treatment	6.47	3.21	5.29,7.65	8.41	3.12	7.09,9.73	0.61*			
Exercise (BSA questionnaire)^c^								2.51	1.41,3.61	0.82***
Pre-treatment	1.82	1.82	1.22,2.42	1.72	1.82	1.12,2.33				
week 9	4.68	1.80	4.00,5.35	1.74	1.79	1.04,2.45	1.64***			
Post-treatment	4.12	1.80	3.46,4.78	1.53	1.77	0.78,2.27	1.45***			
	**Exploratory Analysis (*N*** **= 47)**									
	**Intervention Group** **(*N*** **= 23)**			**Control Group** **(*N*** **= 24)**				**Change from Baseline in Intervention Group Compared With Control Group (*N*** **= 47)**		
Depression (single and with comorbid anxiety disorders) (SCL-90-R)^d^								−0.55	−1.01,−0.09	0.78*
Pre-treatment	1.84	0.79	1.51,2.17	2.00	0.78	1.68,2.32				
Post-treatment	1.10	0.77	0.77,1.44	1.81	0.71	1.42,2.20	0.95**			

*Note*. *SCL-90-R* Symptom Checklist-90-Revised, *PSQI* Pittsburgh Sleep Quality Index, *BSA questionnaire* Exercise Activity Index of the Physical Activity, Exercise, and Sport Questionnaire

^a^ Cohen’s d for post- and intermediate-treatment effect.

^b^ Cohen’s d for the interaction effect.

^c^ Log-transformed data due to a skewed data distribution.

^d^ Participants with depression with and without comorbidities.

*p < .05. **p < .01. ***p < .001

The interaction effect of group by time was significant for the mean amount of exercise, *F* (2, 115) = 16.50, *p* < .001, 95%CI [1.41, 3.61], with larger increases in the intervention group. Bonferroni-corrected intermediate-treatment difference effect, *t* (167) = − 5.83, *p* < .001, and post-treatment difference effect, *t* (170) = − 5.04, *p* < .001, were significant, with higher scores in the intervention group.

Compared to participants of the control group, more participants of the intervention group revealed clinically significant changes of symptomatology, without reaching statistical significance (*χ*^*2*^ (1) = 3.61, *p* = .058.

In a subgroup of participants with depressive disorders, the interaction effect was significant for depression, *F* (1, 37.48) = 5.74, *p* = .022, 95%CI[− 1.01, − 0.09], with larger decreases in the intervention group. The Post-treatment difference in this subgroup was significant, *t* (75) = 2.75, *p* = .008, with lower scores in the intervention group (see Table 3). Across both groups, the change of the mean amount of exercise from pre- to post-treatment assessment significantly predicted the change of global symptom severity, *b* = .35, *t* (49) = 2.61, *p* = .012]. Predictions from the change of exercise on the change of disorder-specific symptoms across both groups and for each group are displayed in Additional file 3.

## Discussion

This RCT compared a group exercise intervention with a passive control group among 72 inactive outpatients, suffering from one or more diagnoses of depressive disorders, anxiety disorders, insomnia, and ADHD. Compared to the control group, the intervention was efficacious in improving global symptom severity, depression, anxiety and sleep quality as well as the amount of exercise with moderate to large effect sizes. Post-treatment difference effects were moderate to large on depression, sleep quality and the amount of exercise. Among participants diagnosed with depressive disorders, the antidepressant effect of the exercise intervention was larger compared to the entire mixed sample. Across both groups, an increase in the amount of exercise predicted the reduction of global symptom severity, indicating that those patients who engaged in more exercise showed decreased symptom severity.

### Efficacy of the intervention

Beneficial effects of the intervention on global symptom severity suggest that the exercise intervention efficaciously reduced symptoms across the included heterogenous sample. On the one hand, there are few studies that investigated the efficacy of exercise among outpatients with heterogenous psychiatric diagnoses [75]. On the other hand, the few existing studies assessed physical health, rather than mental health as primary outcomes [85]. Two RCTs assessed quality of life [86] and general mental health [87] as secondary outcome. Their results suggested improvements on quality of life (i.e., physical function score, social function, emotional role) [86] but not on general mental health (i.e., psychological distress and well-being) [87]. To the best of our knowledge, there exists no study investigating the effects of exercise among a sample with heterogenous diagnoses that include a clinical valid and reliable measure to assess global symptom severity. Whereas the intervention group revealed stronger improvements on global symptom severity, compared to the control group, the post-treatment effect between both groups was not significant. Since sample size calculation was based on large effects of exercise on disorder-specific symptoms among included disorders, power might have been to small to detect treatment effects on global symptom severity across disorders at post-treatment assessment.

Interaction effects of the current intervention on global symptom severity are similar to those of recent meta-analyses [27, 28, 29, 30, 31], evaluating the efficacy of transdiagnostic psychological treatments among patients with depressive disorders and/or anxiety disorders over comparison or control interventions (i.e., diagnosis-specific intervention control, treatment-as-usual, or a waitlist control). Results of these previous studies revealed moderate to large effects on clinical measures assessing symptom severity across included disorders (i.e., depression-anxiety scales, quality of life).

Supporting the assumption of the transdiagnostic efficacy of the intervention, our results demonstrated that the intervention improved one underlying process across included disorders with large effect size: poor sleep quality [21, 22]. This result is similar to a recent meta-analysis of RCTs [34] which demonstrated large beneficial effect of exercise on sleep quality among people with various psychiatric disorders. Nonetheless, the analysis included primarily RCTs assessing the effects of exercise on sleep quality among study samples with specific diagnoses. Only one RCT [88] included participants with a primary diagnosis of a depressive disorder with and without comorbid anxiety disorders. Similarly, another recent meta-analysis [89] that investigated the effects of exercise on various underlying processes across psychiatric disorders, mostly included participants with specific diagnoses or even non-clinical samples. Thus, the findings of our study therefore expand on the results of recent meta-analyses by demonstrating effects on one underlying process across a clinical sample with heterogenous psychiatric disorders.

Correspondingly, our results suggest disorder-specific efficacy by improving symptoms of depression, anxiety and insomnia. The moderate antidepressant effect of the intervention at post-treatment assessment is smaller compared to prior meta-analytical findings suggesting a large antidepressant effect of exercise, over non-active controls [36]. In contrast to our study, prior studies included samples with a primary diagnosis of a depressive disorder only [36]. Although the large antidepressant effect among patients with a primary depressive disorder have been consistently demonstrated in prior studies [35, 36], to the best of our knowledge, the antidepressant effect of exercise among anxiety disorders, insomnia, and ADHD has not been investigated yet. When analyzing the antidepressant efficacy of our exercise intervention among the subsample with a primary diagnosis of a depressive disorder, the effect size is comparable to prior studies [36]. The mean change of anxiety symptoms among the intervention group across the entire mixed sample was large, compared to the control group. Prior exercise trials have reported moderate interaction effects of exercise on anxiety symptoms among patients with anxiety disorders, over non-active controls [38]. This result may suggest that the current exercise intervention might have been more efficacious in reducing anxiety across heterogenous disorders than prior exercise trials that included only anxiety disorders [38]. However, this assumption needs to be considered with caution due to different sample characteristics and exercise modalities.

The post-treatment difference effect between the intervention group and control group on anxiety was not statistically significant. As stated above, the power analysis was based on the median effect size for disorder-specific effects of exercise across depressive disorders, anxiety disorders, and insomnia. Beneficial effects of exercise on anxiety seem to be smaller than those for depression [35] and insomnia symptoms [39]. Furthermore, it appears that effect sizes differ across different anxiety disorders [37]. Therefore, the a-priori determined sample size might have been underestimated to detect treatment effects on anxiety across the heterogenous sample. The large post-treatment effect of exercise on sleep quality among the intervention group, compared to the control group, is comparable to prior meta-analytical findings investigating the effect of exercise on sleep quality in patients with insomnia, over non-active controls [40]. In comparison to meta-analytical findings investigating disorder-specific effects of CBT, over non-active controls, our results showed similar efficacy for depression among depressed participants [90] and sleep quality among the entire mixed sample [91].

The efficacy on global symptom severity and underlying mechanisms across the sample as well as disorder-specific efficacy of the current exercise intervention, suggests that exercise might be able treat a broad range of heterogenous diagnoses with and without comorbidities. Our study results further suggest, that exercise interventions do not necessarily be tailored to a specific psychiatric disorder referring to exercise modalities (i.e., type, frequency) to efficaciously treat disorder-specific symptoms or underlying processes across disorders. Rather, exercise modalities, that have shown therapeutic efficacy among single psychiatric disorders (i.e., two to three times per week, for 10 weeks, at a minimum of moderate intensity and a duration of 30 min, partially supervised or non-supervised, solely aerobic or aerobic combined with resistance training [33, 35, 37, 43]) seem to be adoptable to the treatment of heterogenous diagnoses with and without comorbidities. Similar to the efficacy of transdiagnostic psychological interventions, this may result in a faster and easier dissemination, compared to disorder-specific treatments, because there is no need to learn and apply multiple treatment protocols for different specific disorders [12]. Thus, exercise interventions may improve the existing treatment gap in mental health care [11] by offering an efficacious and effective treatment.

### Effects on exercise behavior

Study results demonstrated that the current exercise intervention was highly efficacious in increasing the amount of exercise, even when participants were not supervised. This is in line with a recent systematic review [46] and Editorial [48], suggesting that exercise interventions, combined with BCTs, are efficacious in increasing the amount of exercise. To date, there are only a few exercise interventions for individuals with psychiatric disorders integrating BCTs [46, 85] and only approximately one quarter of those seem to efficaciously increasing participant’s amount of exercise [85]. The frequent failure to increase exercise behavior among patients with psychiatric disorders may be related to a lack of motivation and exercise-related self-regulatory skills (i.e., volition) in this population [49]. A large proportion of outpatients in Germany do not exercise on a regular base [92]. Thus, the integrated BCTs in our intervention seem to be adequately tailored to outpatients to improve their deficiencies in motivation and volition regarding exercise.

Results of our explorative analyses demonstrated a prediction of symptom reduction by an increase of the performed amount of exercise, indicating that the change of exercise might indeed be one specific mode of action of the therapeutic effects. This moderation effect is often assumed in the exercise literature, however only very few trials do report such effects. As far as we know, only one of the recently published high quality RCTs [45] did show changes of exercise as the specific mode of action of the effects of exercise interventions. In addition to the increase of exercise, results suggest also a maintenance of exercise behavior because participants were still exercising even when they were not supervised. Since there is a lack of follow-up studies of exercise interventions or an absence of measures for exercise behavior, little is known about the maintenance of exercise behavior due to the conduction of exercise interventions combined or without BCTs [85]. As mentioned in the introduction, beneficial effects of exercise seem to be maintained only when exercise behavior has changed sustainably [45]. Since participants were still exercising during the non-supervised period of the intervention, participants might have integrated regular exercise into their daily life routines. Since the amount of exercise was assessed 5 weeks and 2 months after the supervised period, no general conclusions can be drawn about the long-lasting exercise behavior.

### Feasibility of the intervention

Results of this study not only suggest efficacy of the current exercise intervention but also its feasibility in a realistic outpatient setting. First, the study sample included a realistic outpatient sample, waiting for psychotherapeutic treatment, with heterogenous, highly prevalent psychiatric disorders with and without comorbid diagnoses. Second, approximately half of the participants of the intervention group revealed clinically relevant changes of global symptom severity, compared to less than one quarter of the control group. Third, the dropout quote among participants of the intervention group was low. Additionally, only 5.2% (*n* = 2) of the intervention group dropped out due to lost to follow up. Hence, a strong acceptance and few adherence issues regarding the intervention can be assumed. The dropout rate is similar to prior exercise interventions [38, 93] and lower than other health behaviour change interventions (e.g., exercise, health education) for individuals with psychiatric disorders, in which the medium average dropout rate was 45% [94]. The current dropout rate was lower than those of individual and group CBT interventions (35%). Most dropouts were reported in outpatient settings [95]. The lower dropout rate in the current exercise intervention over CBT, might suggest equivalent acceptance of exercise than CBT among outpatients. Forth, the latency between pre-treatment assessment and initiation of the intervention was low, compared to waiting times for psychotherapy of approximately 5 months in Germany [10]. As stated in the introduction, prolonged waiting times for treatment are associated with worsening and chronicity of symptoms and the development of comorbid diagnoses. Consequently, the current intervention could be conducted to improve negative consequences of delayed treatment in Germany. Lastly, in comparison to an average duration of 19 weeks of prior exercise intervention for patients with psychiatric disorders [96], the current exercise intervention was short (12 weeks), including a very short supervised period of 4 weeks. The total number of 12 supervised sessions is equivalent to the average treatment duration of CBT among depressive disorders and anxiety disorders [97, 98].

Hence, in addition to the suggested transdiagnostic and disorder-specific efficacy of the intervention, current results demonstrated that the exercise intervention might be feasible treatment among outpatients, that were waiting for psychotherapeutic treatment in German health care settings in outpatient units and practices.

### Limitations

One limitation of the current study is the use of a passive control group, which does not allow to control for non-specific effects of therapy, such as relationship building or social support. However, the results of our explorative analyses demonstrated a prediction of symptom reduction by an increase of the performed amount of exercise, indicating that the change of exercise might indeed be one specific mode of action of the therapeutic effects. Second, the measure for global symptom severity symptoms was comprised of symptoms that were not characteristic for all included disorders (e.g., aggression). Therefore, the validity of this metric to assess global symptom severity among the current sample is disputable. However, the GSI is a reliable and valid instrument to assess clinical global symptom severity across heterogenous psychiatric disorders and allows to rate clinically relevant changes of symptomatology [99]. Therefore, the use of this measure was reasonable in this study because of the inclusion of heterogenous and highly comorbid psychiatric disorders. Furthermore, the internationally widespread use of this measure [58] allows to compare study results with international publications on the effect of various treatments on symptom severity. Third, eligibility criteria of the current study allowed for a wide age range. However, participants had a low average age, which might limit the generalizability of results to older individuals. Large standard deviations around the mean value, a large age range (19–63 years) of the current sample as well as a similar average age of prior exercise trials [36] might increase the generalizability of results to older individuals. Forth, although the SCID was conducted at pre-treatment and post-treatment assessment, we did not include results of the SCID from post-treatment assessment in our analyses. We intended to assess clinically relevant changes of symptomatology. If a diagnosis, that was present at pre-treatment assessment, had still been present at post-treatment assessment, relevant changes on symptomatology would not have been able to assess. Therefore, we considered self-reported outcomes (GSI of the SCL-90-R) as a more valid measure to assess clinically relevant changes of symptomatology. Moreover, one eligible participant could meet multiple inclusion diagnoses. Thus, the comparison of the number of inclusion diagnoses between pre-treatment and post-treatment assessment did not seem as a valid indicator of treatment responders vs. non-responders. Alternative indicators of treatment responders vs. non-responders, resulting from analyses of the SCID (e.g., counting of symptom criteria for depressive disorders) seem to be an arbitrary and not valid approach.

### Strengths

First, the high methodological standard is an important strength of the study. The study involved an RCT design with stratified block-randomization, which is considered as the gold standard to evaluate intervention efficacy [100]. Equal treatment arms and allocation maximized internal validity [101]. Strict inclusion and exclusion criteria controlled for factors that may obfuscate outcome measures [102]. Second, the inclusion of a heterogeneous sample with a broad age range and various psychiatric disorders allowed for a high generalizability of the results. The conduction of the intervention in a realistic outpatient setting, the large number of included patients with severe, clinically raised symptoms and comorbid presentations of psychiatric disorders suggest a valid representation of the clinical reality [12].

## Conclusions

In conclusion, our findings suggest the transdiagnostic efficacy of exercise across heterogenous psychiatric disorders with comparable effects to transdiagnostic psychological interventions. The transdiagnostic efficacy of the group exercise intervention “ImPuls”, tailored to and conducted with heterogenous psychiatric disorders, is comparable to disorder-specific exercise interventions or established treatments, such as CBT. The increase of exercise behavior seemed to be responsible for the therapeutic effects of the intervention. The low dropout rate, the short latency from first meeting to intervention initiation, the small number of supervised sessions, and the successful increase and maintenance of exercise by integrating BCTs, may indicate a high feasibility and acceptance of the current exercise intervention. Due to the transdiagnostic efficacy and its feasibility within a real-world outpatient setting, the current exercise intervention may represent a treatment option that could improve the existing treatment gap in the outpatient mental health care in Germany. Future research is required to replicate findings with an active control condition, among older individuals, and additional measures of global symptom severity. A follow-up study will allow to assess the maintenance of treatment effects.

## Supplementary Information

**Additional file 1.** Characteristics of treatment completers versus dropouts.

**Additional file 2.** Sensitivity Analysis using Study Completers.

**Additional file 3.** Additional Explorative Analyses of the Predictive Value of Exercise on Primary Outcomes.

**Additional file 4.** Linear mixed models excluding potential outliers.

## Data Availability

The datasets generated and analyzed during the current study are available in the PsychArchives repository, 10.23668/psycharchives.4625.

## Abbreviations

DALYS: Disability-adjusted life years
OCD: Obsessive compulsive disorders
PTSD: Posttraumatic-stress disorder
ADHD: Attention deficit hyperactivity disorder
CBT: Cognitive behavioural therapy
RCT: Randomized controlled trial
BCT: Behaviour change technique
CONSORT: Consolidated Standards of Reporting Trials
GAF: Global Assessment of Functioning Scale
SCID: Structured Clinical Interview for DSM-IV (SCID)
HASE: Homburg ADHD Scales for Adults
PSQI: Pittsburgh Sleep Quality Index
GSI: Global Severity Index
SCL-90-R: Symptom Checklist-90-Revised
BSA questionnaire: Physical Activity, Exercise, and Sport Questionnaire
SF-B: Sleep Questionnaire B-Revised version
PSS: Perceived Stress Scale
SF-36: 36-Item Short Form Health Survey
SKK-Scale: Self Concordance of Sport- and Exercise-related Goals Scale
RPE Scale: Rating of Perceived Exertion Scale
IBM SPSS: Statistical Package for Social Science
ITT: Intention-to-treat
MCAR: Missing completely at random
