# Supplementary material for: Transdiagnostic efficacy of a group exercise intervention for outpatients with heterogenous psychiatric disorders: a randomized controlled trial
Source: BMC Psychiatry. 2021 Jun 22;21:313. doi: 10.1186/s12888-021-03307-x (PMC8218400; doi:10.1186/s12888-021-03307-x)
Supplement: Supplementary file 1 — Additional file 1. Characteristics of treatment completers versus dropouts. [file 12888_2021_3307_MOESM1_ESM.docx]

## Additional File 1. Characteristics of Treatment Completers Versus Dropouts

**Table A1.** Characteristics of treatment completers versus dropouts

|  | **Completers** | | **Dropouts** | | **Completers Compared With Dropouts (N=72)** | | |
| --- | --- | --- | --- | --- | --- | --- | --- |
| **Measure** | **(N=51)** | | **(N=21)** | |  |  |  |
|  | N | % | N | % | χ² | df | p |
| Female | 37 | 72.5 | 15 | 71.4 | 0.01 | 1 | .923 |
| Married or partnered | 34 | 66.7 | 15 | 71.4 | 0.16 | 1 | .694 |
| High school | 40 | 78.4 | 16 | 76.2 |  |  | 1.000^g^ |
| Employment^a^ | 47 | 95.9 | 17 | 81.0 | 4.20 | 1 | .062 |
| Diagnosis |  |  |  |  |  |  | .585^g^ |
| Depression (single)^b^ | 18 | 35.3 | 6 | 28.6 |  |  |  |
| Depression (comorbid with  anxiety disorders)^c^ | 16 | 31.4 | 7 | 33.3 |  |  |  |
| Panic disorder | 1 | 2.0 | 3 | 14.3 |  |  |  |
| Social anxiety disorder | 2 | 3.9 | 2 | 9.5 |  |  |  |
| Specific anxiety disorder | 2 | 3.9 | 0 | 0.0 |  |  |  |
| Generalized anxiety disorder | 1 | 2.0 | 0 | 0.0 |  |  |  |
| Agoraphobia | 1 | 2.0 | 1 | 4.8 |  |  |  |
| Obsessive-compulsive disorder | 0 | 0.0 | 0 | 0.0 |  |  |  |
| Obsessive-compulsive disorder  and social anxiety disorder | 2 | 3.9 | 0 | 0.0 |  |  |  |
| Post-traumatic stress disorder | 3 | 5.9 | 0 | 0.0 |  |  |  |
| Primary insomnia | 4 | 7.8 | 2 | 9.5 |  |  |  |
| Attention deficit hyperactivity  disorder | 1 | 2.0 | 0 | 0.0 |  |  |  |
| Clinically raised symptoms (SCL-90-R, PSQI) | 49 | 98.0 | 21 | 100.0 |  |  | 1.000^g^ |
| Depression^d^, anxiety^d^, insomnia^e^ | 26 | 51.0 | 12 | 57.1 | 0.23 | 1 | .634 |
| Receiving psychiatric drugs^f^ | 27 | 52.9 | 11 | 55.0 | 0.02 | 1 | .876 |
|  | Mean | SD | Mean | SD | t | df | p |
| Age (years) | 35.71 | 12.97 | 36.14 | 14.51 | -0.12 | 33.85 | .905 |
| Global Severity Index (SCL-90-R) | 1.04 | 0.60 | 1.05 | 0.67 | -0.09 | 33.75 | .927 |
| Sleep Quality (PSQI) | 9.12 | 3.43 | 9.24 | 3.52 | -0.13 | 36.77 | .897 |

*Note.* SCL-90-R = Symptom Checklist-90-Revised. PSQI = Pittsburgh Sleep Quality Index.

^a^ The number of participants was *n*= 49 for completers and *n*= 21 for dropouts because of incomplete data at pre-treatment assessment.

^b^ Depressive disorders (single) includes all participants diagnosed with a single depressive disorder.

^c^ Depressive disorders (comorbid with anxiety disorders) includes all participants diagnosed with depressive disorder and comorbid anxiety disorders.

^d^ Depression and anxiety levels were classified as clinically raised when t-values of the sub-scales depression and anxiety of the SCL-90-R were greater or equal to 60 (reference group: healthy men, aged between 35-44).

^e^ Symptoms of insomnia were classified as clinically raised when the cut-off value of five of the total sum score of the PSQI were reached. For clinically raised symptoms and sleep quality, the number of participants was 50 for completers and 21 for dropouts because of incomplete data at pre-treatment assessment on the PSQI.

^f^ For receiving psychiatric drugs, the number of participants was *n*= 51 for completers and *n*= 20 for dropouts because of incomplete data at pre-treatment assessment.

^g^ Fisher’s exact test was used as a replacement for the chi-square test because the frequency of one or more cells was less than 5.
