# Supplementary material for: Transdiagnostic efficacy of a group exercise intervention for outpatients with heterogenous psychiatric disorders: a randomized controlled trial
Source: BMC Psychiatry. 2021 Jun 22;21:313. doi: 10.1186/s12888-021-03307-x (PMC8218400; doi:10.1186/s12888-021-03307-x)
Supplement: Supplementary file 2 — Additional file 2. Sensitivity Analysis using Study Completers. [file 12888_2021_3307_MOESM2_ESM.docx]

## **Additional File 2. Sensitivity Analysis using Study** Completers

Sensitivity analyses examined all outcomes among participants that completed all assessments (per-protocol sample; *n*= 51 (70.8%)). Results of all sensitivity analyses of primary and secondary outcomes and subgroup analysis, including effect sizes, are presented in Table S2.

### ***Primary and Secondary Outcomes***

The interaction effect was significant for global symptom severity, *F*(1,51)= 8.94, *p*= .004, 95%CI[-0.53,-0.11], depression, *F*(1,51)= 7.21, *p*= .010, 95%CI[-0.87,-0.13], anxiety, *F*(1,51)= 10.55, *p*=.002, 95%CI[-0.37,-0.09], and sleep quality, *F*(1,51)= 11.49, *p*= .001 95%CI[-4.90,-1.27], with larger decreases in the intervention group (IG). Post-treatment difference effects for global symptom severity, *t*(66)= 1.05, *p*= .296, depression, *t*(73)= 1.70, *p*= .094, anxiety, *t*(70)= 1.57, *p*= .121, and sleep quality, *t*(83)= 1.90, *p*= .061, were small to moderate with lower scores in the IG, without reaching significance. The interaction effect was significant for the mean amount of exercise, *F*(2,101)= 11.36, *p*< .001, 95%CI[1.07,3.32], with larger increases in the IG. Bonferroni-corrected intermediate-, *t*(124)= -4.80, *p*< .001, and post-, *t*(124.00) = -5.31, *p*< .001.

### ***Explorative Analyses***

In participants with depressive disorders, the interaction effect was significant for depression, *F*(1,34)= 4.83, *p*=.035, 95%CI[-0.98,-0.04], with larger decreases in the IG. The Post-treatment difference in this subgroup was significant, *t*(52)= 2.74, *p*= .008, with lower scores in the IG.

### ***Discussion***

Sensitivity analyses indicated no major differences to the presented intention-to-treat analysis (ITT) on the anxiety scale, the global sleep quality score and the amount of exercise. No major differences were indicated for the subgroup analysis of participants diagnosed with a single depressive disorder (Table S2). The post-treatment difference effect size of the global symptom severity score, changed from moderate (ITT) to small in the sensitivity analysis. The moderate post-treatment difference effect size of the depression score changed from significant (ITT) to non-significant in the sensitivity analyses. The moderate post-treatment difference effect size of the global sleep quality score changed from significant (ITT) to non-significant in the sensitivity analyses. The large post-treatment difference effect size of the depression score among the subgroup of patients with depressive disorders, increased by 0.04 in the sensitivity analysis.

First, sensitivity analyses suggest that the IG and CG differed less on the global symptom severity score at post-treatment assessment among the per-protocol sample compared to the ITT sample. This finding might be explained by different reasons for dropout between IG and CG. The CG dropped out more frequently due to treatment change. Therefore, it can be assumed that the need for treatment was greater in the CG due to an unchanged or increased symptom severity compared to the IG. As a result, participants of the CG with unchanged or increased symptom severity might not have been included in the sensitivity analysis which may have led to more equal outcomes between both groups. Second, the non-significant post-treatment difference effect on depression and sleep quality might be explained by a lack of statistical power.

**Table AS2. Sensitivity Analysis**

|  | **Intervention Group**  **(N=29)** | | |  | **Control Group  (N=22)** | | |  |  | **Change from Baseline in Intervention Group Compared With Control Group (N=51)** | | |
| --- | --- | --- | --- | --- | --- | --- | --- | --- | --- | --- | --- | --- |
|  |  |  |  |  |  |  |  |  |  |  |  |  |
|  |  |  |  |  |  |  |  |  |  |  |  |  |
| **Measure and  Assessment Point** | **Mean** | **SD** | **95% CI** |  | **Mean** | **SD** | **95% CI** | **d^a^** |  | **B** | **95% CI** | **d^b^** |
| Global Severity Index (SCL-90-R) |  |  |  |  |  |  |  |  |  | -0.32 | -0.53,-0.11 | 0.84** |
| Pre-treatment | 1.10 | 0.57 | 0.89,1.31 |  | 0.96 | 0.57 | 0.72,1.20 |  |  |  |  |  |
| Post-treatment | 0.70 | 0.54 | 0.49,0.91 |  | 0.87 | 0.57 | 0.63,1.11 | 0.30 |  |  |  |  |
| Depression (SCL-90-R) |  |  |  |  |  |  |  |  |  | -0.32 | -0.87,-0.13 | 0.75** |
| Pre-treatment | 1.66 | 0.81 | 1.36,1.96 |  | 1.56 | 0.82 | 1.22,1.91 |  |  |  |  |  |
| Post-treatment | 1.01 | 0.81 | 0.71,1.31 |  | 1.41 | 0.82 | 1.06,1.76 | 0.49 |  |  |  |  |
| Anxiety (SCL-90-R)^c^ |  |  |  |  |  |  |  |  |  | -0.23 | -3.37,-0.09 | 0.91** |
| Pre-treatment | 0.59 | 0.33 | 0.47,0.71 |  | 0.51 | 0.33 | 0.37,0.66 |  |  |  |  |  |
| Post-treatment | 0.35 | 0.33 | 0.23,0.48 |  | 0.50 | 0.33 | 0.36,0.65 | 0.45 |  |  |  |  |
| Sleep Quality (PSQI) |  |  |  |  |  |  |  |  |  | -3.078 | -4.90,-1.27 | 0.95** |
| Pre-treatment | 9.69 | 3.26 | 8.48,10.90 |  | 8.40 | 3.32 | 6.99,9.81 |  |  |  |  |  |
| Post-treatment | 6.48 | 3.26 | 5.28,7.69 |  | 8.27 | 3.26 | 6.89,9.66 | 0.55 |  |  |  |  |
| Exercise (BSA-F)^c^ |  |  |  |  |  |  |  |  |  | 2.19 | 1.07,3.31 | 0.77*** |
| Pre-treatment | 2.00 | 1.79 | 1.34,2.66 |  | 1.44 | 1.79 | 0.69,2.20 |  |  |  |  |  |
| week 9 | 4.74 | 1.82 | 4.07,5.41 |  | 1.70 | 1.79 | 0.95,2.46 | 1.68*** |  |  |  |  |
| Post-treatment | 4.19 | 1.79 | 3.53,4.84 |  | 1.44 | 1.79 | 0.69,2.20 | 1.53*** |  |  |  |  |
| **Explorative Analysis (N=26)** | | | | | | | | | | | | |
|  | **Intervention Group**  **(N=16)** | | |  | **Control Group  (N=10)** | | |  |  | **Change from Baseline in Intervention Group Compared With Control Group (N=26)** | | |
| Depression (single and with comorbid anxiety disorders) (SCL-90-R)^d^ |  |  |  |  |  |  |  |  |  | -0.51 | -0.98,-0.04 | 0.75* |
| Pre-treatment | 1.83 | 0.77 | 1.49,2.17 |  | 2.08 | 0.77 | 1.65,2.51 |  |  |  |  |  |
| Post-treatment | 1.10 | 0.77 | 0.76,1.43 |  | 1.86 | 0.77 | 1.43,2.29 | 0.99** |  |  |  |  |

*Note.* SCL-90-R = Symptom Checklist-90-Revised. PSQI = Pittsburgh Sleep Quality Index. BSA questionnaire = Exercise Activity Index of the Physical Activity, Exercise, and Sport Questionnaire.

^a^ Cohen’s d for post- and intermediate-treatment effect.

^b^ Cohen’s d for the interaction effect.

^c^ Log-transformed data due to a skewed data distribution.

^d^ Participants with depression with and without comorbidities.

*p<.05. **p<.01. ***p<.001
