# Supplementary material for: Transdiagnostic efficacy of a group exercise intervention for outpatients with heterogenous psychiatric disorders: a randomized controlled trial
Source: BMC Psychiatry. 2021 Jun 22;21:313. doi: 10.1186/s12888-021-03307-x (PMC8218400; doi:10.1186/s12888-021-03307-x)
Supplement: Supplementary file 3 — Additional file 3. Additional Explorative Analyses of the Predictive Value of Exercise on Primary Outcomes. [file 12888_2021_3307_MOESM3_ESM.docx]

## Additional File 3. Additional Explorative Analyses of the Predictive Value of Exercise on Primary Outcomes

Overall, the change of the mean amount of exercise from pre-treatment to post-treatment assessment significantly predicted the change of the mean depression score, *b*= .39, *t*(49)= 2.99, *p*= .004, the change of the mean anxiety score, *b*= .41, *t*(49)= 2.99, *p*= .003, and the change of the mean global sleep quality score, *b*= .31, *t*(48)=2.23, *p*= .030.

Among participants of the IG, the prediction was significant for the change of the mean depression score, *b*= .37, *t*(27)= 2.09, *p*= .046, and the change of the mean anxiety score, *b*= .38, *t*(27)= 2.16, *p*= .040, but not significant for the change of the global score of symptom severity, *b*= .32, *t*(27)= 1.77, *p*= .089, and the sleep quality score, *b*= .20, *t*(27)=1.04, *p*= .310.

Among participants of the CG, the prediction was neither significant on the change of the global symptom severity score, *b*= .07, *t*(20)= 0.31, *p*= .757, nor on the change of the mean depression score, *b*= .21, *t*(20)= 0.96, *p*= .349, nor on the change of the mean anxiety score, *b*= .15, *t*(20)=0.66, *p*= .514, nor on the change of the mean subjective sleep quality score, *b*= .12, t(19)=0.52, *p*= .610.
