# Supplementary material for: Transdiagnostic efficacy of a group exercise intervention for outpatients with heterogenous psychiatric disorders: a randomized controlled trial
Source: BMC Psychiatry. 2021 Jun 22;21:313. doi: 10.1186/s12888-021-03307-x (PMC8218400; doi:10.1186/s12888-021-03307-x)
Supplement: Supplementary file 4 — Additional file 4. Linear mixed models excluding potential outliers. [file 12888_2021_3307_MOESM4_ESM.docx]

## Additional File 4. Linear mixed models excluding potential outliers

|  | **Intervention Group**  **(N=32)** | | |  | **Control Group**  **(N=30)** | | |  |  | **Change from Baseline in Intervention Group Compared With Control Group (N=72)** | | |
| --- | --- | --- | --- | --- | --- | --- | --- | --- | --- | --- | --- | --- |
|  |  |  |  |  |  |  |  |  |  |  |  |  |
|  |  |  |  |  |  |  |  |  |  |  |  |  |
| **Measure and  Assessment Point** | **Mean** | **SD** | **95% CI** |  | **Mean** | **SD** | **95% CI** | **d^a^** |  | **B** | **95% CI** | **d^b^** |
| Global Severity Index (SCL-90-R) |  |  |  |  |  |  |  |  |  | -0.31 | -0.52,-0.11 | 0.84** |
| Pre-treatment | 1.01 | 0.47 | 0.85,1.18 |  | 0.97 | 0.47 | 0.80,1.14 |  |  |  |  |  |
| Post-treatment | 0.61 | 0.45 | 0.44,0.79 |  | 0.88 | 0.44 | 0.69,1.06 | 0.59* |  |  |  |  |
| Depression (SCL-90-R) |  |  |  |  |  |  |  |  |  | -0.50 | -0.86,-0.14 | 0.75** |
| Pre-treatment | 1.54 | 0.73 | 1.29,1.80 |  | 1.55 | 0.73 | 1.29,1.82 |  |  |  |  |  |
| Post-treatment | 0.89 | 0.71 | 0.62,1.16 |  | 1.40 | 0.70 | 1.11,1.70 | 0.73* |  |  |  |  |
| Anxiety (SCL-90-R)^c^ |  |  |  |  |  |  |  |  |  | -0.24 | -0.38,-0.10 | 0.94** |
| Pre-treatment | 0.58 |  | 0.46,0.69 |  | 0.53 |  | 0.41,0.65 |  |  |  |  |  |
| Post-treatment | 0.32 |  | 0.20,0.44 |  | 0.52 |  | 0.39,0.64 | 0.61* |  |  |  |  |
| Sleep quality (PSQI) |  |  |  |  |  |  |  |  |  | -3.32 | -4.69,--1.95 | 1.34*** |
| Pre-treatment | 9.36 | 2.99 | 8.33,10.40 |  | 8.47 | 2.97 | 7.45,9.48 |  |  |  |  |  |
| Post-treatment | 5.88 | 2.84 | 4.78,6.99 |  | 8.30 | 2.73 | 7.15,9.45 | 0.90** |  |  |  |  |
| *Note.* SCL-90-R = Symptom Checklist-90-Revised.  ^a^ Cohen’s d for post-treatment effect.  ^b^ Cohen’s d for the interaction effect.  ^c^ Log-transformed data due to a skewed data distribution.  *p<.05. **p<.01. ***p<.001. | | | | | | | | | | | | |
